# Strategic Planning Perception as a Mediator Between Psychological Resilience and Coping Strategies Among Nursing Staff: Implications for Workforce Resilience

**DOI:** 10.1155/jonm/5563937

**Published:** 2026-08-26

**Authors:** Wafaa Ahmed Saleh, Mona Metwally El-Sayed, Eman Mohamed Ahmed Elshazly, Ahmed Abdellah Othman, Boshra Karem Mohamed El-Sayed, Nada Alqarawi, Aml AbdElaal Mohamed Ali

**Affiliations:** ^1^ Psychiatric Nursing and Mental Health Department, Faculty of Nursing, Qena University, Qena, Egypt; ^2^ Department of Community, Psychiatric, and Mental Health Nursing, College of Nursing, Qassim University, Buraydah 52571, Saudi Arabia, qu.edu.sa; ^3^ Nursing Services Department, Qassim University Medical City, Buraydah 52571, Saudi Arabia; ^4^ Nursing Administration Department, Faculty of Nursing, Qena University, Qena, Egypt; ^5^ Nursing Administration Department, Faculty of Nursing, Sohag University, Sohag, Egypt, sohag-univ.edu.eg; ^6^ Nursing Administration and Education Department, Faculty of Nursing, University of Tabuk, Tabuk, Saudi Arabia, ut.edu.sa

**Keywords:** coping strategies, cross-sectional study, nursing staff, psychological resilience, strategic planning perception, workforce resilience

## Abstract

**Background:**

Nurses frequently encounter demanding clinical environments that require effective coping to maintain performance and well‐being. Psychological resilience has been recognized as a key personal resource that enables nurses to adapt to occupational stress. However, organizational factors, such as nurses’ perceptions of strategic planning, may also shape how resilience translates into effective coping strategies.

**Aim:**

This study aimed to examine the mediating role of strategic planning perception in the relationship between psychological resilience and coping strategies among nursing staff.

**Methods:**

A quantitative cross‐sectional correlational design was employed. A nonprobability convenience sample of 379 nurses working at Qena University Hospital, Egypt, participated in the study. Data were collected using the Strategic Plan Perception Scale, the Connor–Davidson Resilience Scale (CD‐RISC‐10), and the Coping Strategies Inventory Short Form (CSI‐SF). Structural equation modeling (SEM) with path analysis was performed using SPSS and AMOS Version 23 to test the hypothesized mediation model while controlling for demographic and work‐related variables. Bootstrapped confidence intervals were used to assess the significance of indirect effects.

**Results:**

Psychological resilience showed a significant positive association with coping strategies (*β* = 0.312, *p* < 0.001) and strategic planning perception (*β* = 0.521, *p* < 0.001). Strategic planning perception was also positively associated with coping strategies (*β* = 0.213, *p* < 0.001). Mediation analysis revealed that strategic planning perception partially mediated the relationship between psychological resilience and coping strategies (indirect effect = 0.181, 95% CI [0.016, 0.088], *p* < 0.001), with the indirect effect accounting for approximately 37% of the total effect.

**Conclusions:**

Strategic planning perception was identified as an organizational mechanism associated with the relationship between psychological resilience and coping strategies among nursing staff. Both individual psychological resources and organizational planning processes were found to be related to nurses’ responses to workplace stress. These findings suggest that integrated approaches simultaneously strengthening personal resilience and enhancing organizational communication and participatory planning may be beneficial.

**Implications for Nursing Management:**

Nursing leaders may consider strengthening resilience development initiatives and promoting transparent and participatory strategic planning processes. Enhancing nurses’ understanding and involvement in organizational planning may be associated with adaptive coping and contribute to workforce resilience. Future longitudinal research is needed to establish causal relationships and evaluate the effectiveness of integrated interventions targeting both personal and organizational resources.

## 1. Introduction

Nurses play a central role in delivering safe, high‐quality healthcare within increasingly complex and demanding clinical environments. The multifaceted demands of patient care, compounded by persistent organizational pressures, including staffing shortages, high workloads, rapid technological advancements, and systemic changes in health service delivery, create chronic stressors that profoundly affect nurses’ psychological well‐being, professional performance, and retention [1, 2]. Consequently, understanding the factors that enable nurses to maintain psychological stability and effective functioning under persistent stress has become a critical priority for healthcare organizations, nursing leaders, and policymakers committed to workforce sustainability and quality patient care [3–5].

Psychological resilience, defined as the capacity to adapt, recover, and maintain psychological stability in the face of adversity, trauma, or significant stress, has emerged as a fundamental personal resource supporting nurses’ ability to navigate workplace challenges and sustain professional functioning [6, 7]. Resilience is increasingly conceptualized as a multidimensional construct involving not merely the absence of psychopathology following adversity but the active process of positive adaptation, emotional regulation, and cognitive flexibility that enables individuals to transform potential threats into manageable challenges [8, 9].

A substantial and growing body of evidence indicates that resilience contributes positively to nurses’ psychological well‐being, job satisfaction, professional commitment, and retention, while simultaneously mitigating negative outcomes such as burnout, compassion fatigue, and turnover intention [3, 6, 10, 11]. Research has demonstrated that resilient nurses exhibit greater emotional stability, enhanced problem‐solving abilities, and more positive appraisals of workplace stressors, all of which facilitate sustained performance in high‐pressure clinical settings [12–14]. A recent systematic review by Yu et al. [6] synthesized evidence from multiple studies, confirming that resilience levels among nurses are influenced by a combination of individual, professional, and environmental factors, and that higher resilience consistently correlates with reduced burnout and improved job satisfaction across diverse healthcare settings.

Coping strategies encompass the cognitive, emotional, and behavioral efforts individuals employ to manage internal and external demands perceived as stressful or exceeding their available resources [15]. In nursing contexts, coping behaviors play a crucial role in stress management and long‐term professional outcomes, as nurses must continuously navigate emotionally charged situations, ethical dilemmas, and life‐threatening emergencies that demand effective psychological regulation [16–18].

Contemporary literature distinguishes between adaptive coping strategies, such as problem‐focused engagement, active emotional regulation, and seeking social support, and maladaptive strategies, including avoidance, disengagement, and emotional suppression [19, 20]. Among nurses, adaptive coping is consistently associated with lower burnout, reduced psychological distress, and better overall mental health outcomes, whereas maladaptive coping exacerbates stress, impairs work functioning, and increases the risk of professional exhaustion [16, 21]. Evidence further indicates that coping effectiveness is strongly influenced by psychological resilience, which enhances positive stress appraisal and facilitates the selection and implementation of adaptive behavioral responses [9, 12]. Resilient nurses are more likely to engage in problem‐focused coping, reframe challenges constructively, and maintain emotional equilibrium during stressful encounters, thereby preventing the cumulative depletion of psychological resources that characterizes burnout [1, 13, 22]. However, while the direct relationship between resilience and coping has been well‐documented, what remains less clear is how organizational perceptions and contextual factors influence nurses’ adoption of specific coping strategies.

Strategic planning refers to a structured, systematic process of defining long‐term organizational goals, allocating resources, and guiding institutional actions to respond effectively to internal and external environmental changes [23]. In healthcare organizations, effective strategic planning contributes to operational efficiency, organizational adaptability, quality improvement, and the alignment of institutional objectives with frontline clinical realities [24, 25]. Strategic planning processes typically encompass goal setting, resource allocation, performance measurement, and stakeholder engagement, all of which shape the organizational context within which nurses operate [26].

Perceptions of strategic planning reflect how nurses understand, interpret, and evaluate these organizational processes, including their transparency, inclusiveness, coherence, and relevance to daily clinical practice [27]. When nurses perceive strategic planning as transparent, participatory, and aligned with clinical goals, they are more likely to experience enhanced role clarity, organizational trust, and psychological safety [24, 26]. Conversely, when strategic planning is perceived as opaque, top–down, or disconnected from frontline realities, nurses may experience uncertainty, reduced engagement, and diminished organizational commitment [23, 25].

Importantly, positive perceptions of strategic planning may shape how nurses evaluate workplace stressors and select coping responses. Conceptually, when nurses understand organizational goals and perceive themselves as integral to planning processes, they may experience greater control over their work environment, an enhanced sense of purpose, and a stronger identification with institutional objectives, all of which may facilitate adaptive coping [28, 29]. Clear strategic communication and involvement in planning can reduce uncertainty, enhance psychological safety, and promote structured, engagement‐oriented coping responses among staff [25, 30]. Furthermore, nurses who perceive strategic planning as supportive and coherent may be more likely to interpret organizational demands as manageable challenges rather than insurmountable threats, thereby fostering constructive coping behaviors [31, 32].

### 1.1. Theoretical Framework

The current study is grounded in two complementary theoretical perspectives: Conservation of Resources (COR) Theory and Cognitive Appraisal Theory, which together provide a comprehensive framework for understanding how personal and organizational resources interact to shape coping behaviors. COR Theory [33] proposes that individuals strive to obtain, retain, and protect valued resources, whether psychological, social, material, or organizational, to mitigate stress and enhance well‐being. According to this framework, resource loss is a primary driver of stress, while resource gain facilitates positive adaptation and resilience [34, 35]. Within this theoretical perspective, psychological resilience can be conceptualized as a personal resource that enables nurses to withstand adversity and maintain psychological stability, while strategic planning perception represents an organizational resource that enhances nurses’ sense of stability, predictability, and control within the work environment [32, 36]. COR theory further posits that individuals with greater personal resources are better equipped to acquire, recognize, and mobilize additional contextual resources, creating what Hobfoll and Shirom [37] termed “resource caravans,” clusters of resources that travel together and mutually reinforce one another. Thus, resilient nurses may be more capable of perceiving and utilizing strategic planning structures as supportive organizational resources, which in turn facilitate adaptive coping strategies and prevent further resource depletion [22, 35].

Cognitive Appraisal Theory [15] emphasizes the central role of individual appraisal processes in determining how stressors are perceived, interpreted, and managed. According to this framework, individuals engage in primary appraisal (evaluating the significance of a stressor) and secondary appraisal (assessing available coping resources and options), which together shape emotional responses and coping behaviors [31, 32]. Personal dispositions such as resilience influence these appraisal processes, as resilient individuals tend to interpret stressors as manageable challenges rather than overwhelming threats [38]. Organizational perceptions, including strategic planning perception, may similarly shape secondary appraisal by influencing how individuals interpret workplace conditions and evaluate available support structures [28]. When nurses perceive strategic planning processes as coherent, transparent, and aligned with their professional values, they may appraise workplace demands as more manageable and organizational resources as more accessible, thereby facilitating constructive coping responses [25, 31].

Although researchers have extensively examined psychological resilience and coping strategies independently, there remains a limited understanding of the organizational mechanisms that facilitate or hinder the translation of resilience into effective coping behaviors. Most existing studies have focused on direct relationships between resilience and coping, or on interventions designed to enhance resilience, but few have examined the cognitive organizational perceptions that may shape these relationships [39–41]. Importantly, to the best of our knowledge, no published studies to date have explicitly tested whether strategic planning perception mediates the relationship between psychological resilience and coping strategies among nurses. While research has explored related constructs such as perceived organizational support, workplace climate, and leadership styles as moderators or mediators of resilience‐coping relationships [26, 28], the specific role of strategic planning perception remains unexplored. This gap is significant because strategic planning represents a foundational organizational process that shapes nurses’ understanding of institutional direction, resource allocation, and professional expectations [23, 24]. Understanding whether and how perceptions of strategic planning influence coping behaviors could inform more integrated workforce management strategies that leverage both personal and institutional resources to support nurse well‐being and retention.

The present study addresses this gap by testing a mediation model linking psychological resilience, strategic planning perception, and coping strategies among nursing staff. By illuminating this mediating pathway, the research contributes to a more nuanced understanding of individual–organizational interactions in nursing workforce sustainability. Furthermore, identifying strategic planning perception as a mechanism linking resilience with coping behaviors can inform targeted managerial interventions aimed at enhancing both nurse well‐being and organizational effectiveness, thereby addressing the pressing challenges of nurse burnout, turnover, and workforce shortages that confront healthcare systems globally [1, 3]. Thus, this study aimed to assess the relationships among psychological resilience, strategic planning perception, and coping strategies among nursing staff, and to determine whether strategic planning perception mediates the relationship between psychological resilience and coping strategies, with the goal of informing strategies to enhance workforce resilience and sustainability in healthcare organizations.

**FIGURE 1:**
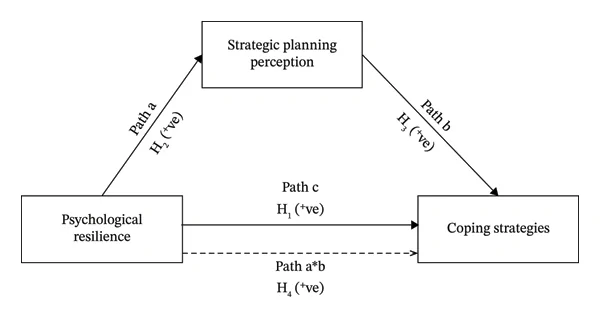
Conceptual model of the study.

Accordingly, the following hypotheses were formulated:

 H1: Psychological resilience is positively associated with adaptive coping strategies among nursing staff.
 H2: Psychological resilience is positively associated with strategic planning perception among nursing staff.
 H3: Strategic planning perception is positively associated with adaptive coping strategies among nursing staff.
 H4: Strategic planning perception mediates the relationship between psychological resilience and coping strategies among nursing staff, such that higher psychological resilience is associated with stronger strategic planning perception, which in turn enhances adaptive coping strategies (Figure 1).

## 2. Methods

### 2.1. Research Design and Settings

A quantitative, cross‐sectional correlational design was employed. The study was conducted at Qena University Hospital in Qena, Egypt. The hospital was purposively selected due to its high patient volume, wide range of medical and surgical specialties, and diverse nursing workforce, providing an appropriate context for investigating factors influencing workforce resilience. The cross‐sectional design is suitable for examining associations among variables at a single time point; however, it limits the ability to establish causal relationships or determine temporal precedence. Accordingly, the analytical framework, path analysis within structural equation modeling (SEM) with bootstrapped confidence intervals, was selected to test the statistical significance of the hypothesized mediation model, recognizing that causal inference cannot be established from cross‐sectional data [42]. The study was designed and reported in accordance with the Strengthening the Reporting of Observational Studies in Epidemiology (STROBE) guidelines to ensure methodological rigor, transparency, and standardized reporting of observational research.

### 2.2. Participants and Sampling

The study population consisted of staff nurses employed at Qena University Hospital. Inclusion criteria were as follows: (1) full‐time employment, (2) voluntary participation, and (3) at least 1 year of clinical experience to ensure sufficient professional exposure and familiarity with nursing roles.

The required sample size was determined using G∗Power Version 3.1.9.7, based on parameters for linear multiple regression. Power analysis indicated that a minimum of 350 participants were needed to detect a medium effect size (*f* ^2^ = 0.15) with a statistical power of 0.85, an alpha level of 0.05, and three predictor variables. To account for potential nonresponse and an estimated 10% attrition rate, the sample size was increased to 379 participants. A nonprobability convenience sampling method was employed to recruit participants, ensuring accessibility and feasibility while maintaining representation of the nursing staff relevant to the study’s objectives.

### 2.3. Study Instruments

Data were collected using three validated self‐reported questionnaires in addition to a demographic and professional characteristics form. Demographic variables included age, gender, educational qualification, current working unit, and years of experience in the nursing profession.

### 2.4. Strategic Plan Perception Scale

It was developed by Aydın et al. [27] to assess nurses’ perceptions of the preparation, implementation, and evaluation of strategic plans. The scale consists of 21 items, each rated on a five‐point Likert scale ranging from 1 (*Strongly Disagree*) to 5 (*Strongly Agree*), yielding total scores between 21 and 105. Higher scores indicate a more positive perception and greater engagement with strategic planning processes. The internal consistency of the scale was excellent, with a Cronbach’s alpha coefficient of 0.97, demonstrating high reliability [27].

### 2.5. Connor–Davidson Resilience Scale (CD‐RISC‐10)

The CD‐RISC‐10 was originally developed by Connor and Davidson [8] to assess psychological resilience as a multidimensional construct reflecting the ability to cope with stress and adversity. The 10‐item version (CD‐RISC‐10) used in this study is a validated brief form derived from the original 25‐item scale, demonstrating strong psychometric properties across diverse populations [43, 44]. The scale consists of 10 items designed as a Likert‐type additive scale with five response options ranging from 0 (“*Never*”) to 4 (“*Almost Always*”), representing a single underlying dimension of resilience. The total score is calculated by summing responses across all items, yielding a range from 0 to 40, with higher scores indicating higher levels of psychological resilience. The scale demonstrates excellent internal consistency, with a Cronbach’s alpha coefficient of 0.93 in previous validation studies [44] and 0.85 in the present study, reflecting high reliability.

### 2.6. Coping Strategies Inventory Short–Form (CSI‐SF)

It was developed by Addison et al. [19] to assess coping thoughts and behaviors in response to stressful situations. The scale consists of 16 items organized into four 4‐item subscales: (a) Problem‐Focused Engagement, (b) Problem‐Focused Disengagement, (c) Emotion‐Focused Engagement, and (d) Emotion‐Focused Disengagement. Responses are rated on a four‐point Likert scale ranging from 1 (“*Never*”) to 4 (“*Always*”), with each subscale yielding scores from 4 to 64. Higher scores indicate a greater use of the respective coping strategy. The internal consistency of the scale is high, with a Cronbach’s alpha coefficient of 0.87, demonstrating good reliability [19].

### 2.7. Instrument Validity and Reliability

A rigorous back‐translation procedure was utilized to translate the original English instruments into Arabic, following the methodological framework outlined by Ozolins et al. [45]. This approach ensured semantic equivalence, cultural relevance, and conceptual accuracy, thereby supporting the validity and reliability of the instruments in the Egyptian healthcare setting. After translation, content validity was evaluated by a panel of five nursing management experts, who assessed the instruments for clarity, relevance, and contextual appropriateness. Minor wording modifications were implemented based on their recommendations to enhance linguistic precision and cultural suitability. Subsequently, a pilot study involving 40 nurses was conducted to assess the clarity, feasibility, and completion time of the instruments. Participants from the pilot study were excluded from the main study to prevent potential bias in the final analysis.

Confirmatory factor analysis (CFA) was conducted using IBM SPSS AMOS Version 23 to evaluate the construct validity of the translated instruments. Model fit was assessed based on established criteria: root mean square error of approximation (RMSEA) < 0.08, *χ* ^2^/df < 5, and GFI, CFI, and IFI > 0.90 [46]. The Strategic Plan Perception Scale showed good fit (*χ* ^2^ = 118.031, *χ* ^2^/df = 2.84, GFI = 0.922, IFI = 0.902, CFI = 0.916, RMSEA = 0.071). The CD‐RISC‐10 demonstrated satisfactory fit (*χ* ^2^ = 232.042, *χ* ^2^/df = 2.223, GFI = 0.948, IFI = 0.961, CFI = 0.920, RMSEA = 0.063). The CSI‐SF also met acceptable thresholds (*χ* ^2^ = 253.031, *χ* ^2^/df = 2.182, GFI = 0.934, IFI = 0.929, CFI = 0.936, RMSEA = 0.075). These results confirm the construct validity of all three scales within the study sample.

Factor loadings for all items across the two instruments ranged from 0.52 to 0.89, with all items exceeding the recommended threshold of 0.40 [47]. Modification indices were examined to identify potential areas for model improvement; however, no substantial modifications were required as all models demonstrated acceptable fit. Composite reliability (CR) values for all scales exceeded the recommended threshold of 0.70, indicating satisfactory internal consistency [48]. The Strategic Planning Perception Scale demonstrated a CR of 0.94, the CD‐RISC‐10 showed a CR of 0.87, and the CSI‐SF exhibited a CR of 0.89. Average variance extracted (AVE) values for convergent validity assessment were 0.62, 0.55, and 0.58 for the three scales, respectively, all meeting the recommended threshold of > 0.50 [47]. Discriminant validity was assessed using the Fornell–Larcker criterion, which requires that the square root of the AVE for each construct exceeds the correlations between constructs. The square roots of AVE were 0.79 for Strategic Planning Perception, 0.74 for Psychological Resilience, and 0.76 for Coping Strategies, all exceeding the interconstruct correlations, thereby confirming discriminant validity.

Internal consistency reliability was evaluated using Cronbach’s alpha. The Strategic Planning Perception Scale demonstrated excellent reliability (*α* = 0.96). The CD‐RISC‐10 and the CSI‐SF also exhibited strong internal consistency, with Cronbach’s alpha coefficients of 0.85 and 0.88, respectively. These findings, combined with the composite reliability values, indicate that all three instruments possessed satisfactory reliability and were appropriate for use in the present study.

### 2.8. Ethical Considerations

All procedures followed ethical principles in the Declaration of Helsinki (Oct 2008), ensuring participant protection, dignity, and rights. Ethical approval was received from the Research Ethics Committee of the Faculty of Nursing at Qena University (Approval Number: SVU‐NUR‐PYS‐21‐2‐11‐2024), and administrative approval was obtained from Qena University Hospital before data collection. Participants were thoroughly informed about the study’s aims, procedures, risks, and benefits and provided written consent, affirming voluntary participation. They were told they could withdraw anytime without penalty. Confidentiality was maintained by designing questionnaires without identifying info, assigning unique codes, storing data securely, and reporting findings in aggregate. Clinical responsibilities were never affected, and interactions respected autonomy and professional duties, safeguarding participants’ rights and well‐being.

### 2.9. Data Collection

Data were collected over 5 months using anonymous, self‐administered paper‐based questionnaires distributed to nurses at Qena University Hospital. To minimize interference with patient care, questionnaires were distributed during shift change overlaps (15–20 min), scheduled break times, and monthly staff meetings, with designated quiet areas provided for completion. Trained researchers were present on‐site to explain the study protocol, answer questions, and collect completed questionnaires, reducing the time nurses needed to spend on administrative tasks. Each questionnaire required approximately 15–20 min to complete and included a cover letter explaining the study purpose, voluntary participation, and confidentiality assurances. A secure drop‐box system was placed in each unit’s staff lounge, allowing nurses to complete and return questionnaires anonymously at their convenience without coordinating with research staff. The research team maintained daily communication with nurse managers to ensure data collection did not coincide with periods of high patient acuity, emergencies, or staffing shortages. Questionnaires were designed to exclude any personally identifiable information, and completed forms were stored in locked cabinets accessible only to the research team. Of the 450 questionnaires distributed, 397 were returned (response rate 88.2%), with 379 included in the final analysis after data cleaning (completion rate 84.2%). Participants received verbal appreciation for their contribution, and no incentives were provided. All procedures complied with the Declaration of Helsinki, ensuring participants’ clinical responsibilities were never compromised and all interactions respected their autonomy and professional obligations.

### 2.10. Statistical Analysis

Descriptive statistics, including frequencies, percentages, means, and standard deviations, were used to summarize participants’ sociodemographic and professional characteristics. Preliminary data screening revealed minimal missing data (< 5%), which were handled using listwise deletion as Little’s MCAR test was non‐significant (*χ* ^2^ = 45.23, df = 38, *p* = 0.196). Normality assumptions were assessed through skewness and kurtosis values, which ranged from −0.42 to 0.38 and −0.56 to 0.72, respectively, all within acceptable thresholds (±2 for skewness, ±7 for kurtosis), indicating normally distributed data suitable for maximum likelihood estimation. Multicollinearity was assessed using variance inflation factor (VIF) and tolerance values, with VIF values ranging from 1.12 to 1.89 (well below the threshold of 10) and tolerance values from 0.53 to 0.89 (above 0.10), indicating no serious multicollinearity concerns. Pearson’s correlation coefficient was employed to assess bivariate associations among continuous variables. To test the hypothesized mediation model, a path analysis approach was employed within the SEM framework using IBM SPSS AMOS Version 23. The model was estimated using observed composite scores (total scale scores) rather than latent variables, which is consistent with path analysis with observed variables. The use of SEM terminology is justified as the analysis simultaneously estimated direct, indirect, and total effects, employed bootstrapped confidence intervals for indirect effect testing, and evaluated overall model fit, all standard SEM procedures. The model examined the mediating role of strategic planning perception in the relationship between psychological resilience (independent variable) and coping strategies (dependent variable), controlling for age, gender, marital status, educational level, and years of experience. Model fit was assessed using chi‐square (*χ* ^2^), Comparative Fit Index (CFI), Incremental Fit Index (IFI), and RMSEA, with acceptable fit defined as RMSEA < 0.08, CFI and IFI > 0.90, and *χ* ^2^/df < 5. The significance of the indirect (mediated) effect was determined using bootstrapped 95% confidence intervals based on 5000 bootstrap samples, with mediation considered significant if the confidence interval did not include zero. Both unstandardized (*B*) and standardized (*β*) regression coefficients were reported, along with *R* ^2^ values for endogenous variables to facilitate interpretation of practical significance. A two‐tailed significance level of 0.05 was applied for all statistical tests, and all assumptions of path analysis were assessed and found to be satisfied.

## 3. Results

### 3.1. Participant Demographic and Work‐Related Characteristics

More than half of the participants (52.0%) were younger than 30 years (*M* = 32.66 ± 6.72 years), while 24.3% were aged 30–40 years, 17.4% were aged 41–50 years, and only 6.3% were over 50 years. The majority of participants were female (71.5%), while males constituted 28.5% of the sample. Regarding educational qualifications, 39.6% held a bachelor’s degree in nursing, 38.5% had a technical institute diploma, and 21.9% possessed a master’s degree. The majority of nurses worked in medical units (44.9%), followed by surgical units (31.1%) and critical and intensive care units (24.0%). Nearly half of the participants (48.8%) had 5–10 years of professional experience, while 45.4% had less than 5 years, and only 4.2% and 1.6% had 11–20 years and more than 20 years of experience, respectively (*M* = 8.32 ± 6.52 years), as shown in Table 1.

**TABLE 1:** Distribution of participants according to their demographics and work‐related characteristics (n = 379).

Sociodemographic and work‐related characteristics	No	%
Age		
< 30	197	52.0
30–40	92	24.3
41–50	66	17.4
> 50	24	6.3
Mean ± SD	32.66 ± 6.72	
Gender		
Male	108	28.5
Female	271	71.5
Educational qualifications		
Technical institute diploma in nursing	146	38.5
Bachelor’s degree in nursing	150	39.6
Master’s degree	83	21.9
Working units		
Medical units	170	44.9
Surgical units	118	31.1
Critical and Intensive Care Units	91	24.0
Years of experience in the nursing profession		
< 5	172	45.4
5–10	185	48.8
11–20	16	4.2
> 20	6	1.6
Mean ± SD	8.32 ± 6.52	

Abbreviation: SD, standard deviation.

### 3.2. Descriptive Statistics and Correlations Among Study Variables

The mean scores for the study variables were 73.47 (SD = 13.67) for strategic planning perception, 57.28 (SD = 6.42) for psychological resilience, and 56.35 (SD = 7.99) for coping strategies. All variables were positively and significantly correlated. Strategic planning perception was significantly associated with psychological resilience (*r* = 0.521, *p* < 0.01) and coping strategies (*r* = 0.632, *p* < 0.01). Psychological resilience was also significantly correlated with coping strategies (*r* = 0.697, *p* < 0.05), as presented in Table 2.

**TABLE 2:** Descriptive statistics and pairwise correlations between the study variables (*n* = 379).

Variables	1	2	3
1. Strategic plan perception	1		
2. Psychological resilience	0.521^∗∗^	1	
3. Coping strategies	0.632^∗∗^	0.697^∗^	1
Mean	73.47	57.28	56.35
Standard deviation (SD)	13.665	6.415	7.989

^∗∗^Correlation is significant at the 0.01 level (2‐tailed).

^∗^Correlation is significant at the 0.05 level (2‐tailed).

### 3.3. Mediation Analysis: Testing the Hypothesized Model

SEM with path analysis was conducted to examine the mediating role of strategic planning perception in the relationship between psychological resilience and coping strategies. The model demonstrated acceptable fit to the data (*χ* ^2^ = 32.210, df = 14, *p* < 0.001; CFI = 0.981; IFI = 0.982; RMSEA = 0.059). It should be noted that the CFI and IFI values approached 1.000 (specifically 0.981 and 0.982), which reflects the simplicity of the path model with a limited number of variables and paths. Perfect or near‐perfect fit indices are not uncommon in parsimonious models with few degrees of freedom, as the model is relatively simple, and the specified relationships are well‐supported by the data [49]. The model explained 27.1% of the variance in strategic planning perception (*R* ^2^ = 0.271) and 48.6% of the variance in coping strategies (*R* ^2^ = 0.486), indicating that the predictors accounted for substantial proportions of the variance in the endogenous variables.

Psychological resilience had a significant positive direct effect on coping strategies (*B* = 0.239, *β* = 0.312, SE = 0.063, *t* = 3.799, and *p* < 0.001), Supporting H1. This indicates that for every one‐unit increase in psychological resilience, coping strategies increased by 0.239 units on the unstandardized scale, representing a moderate standardized effect. Psychological resilience also showed a significant positive effect on strategic planning perception (*B* = 0.503, *β* = 0.521, SE = 0.106, *t* = 4.729, and *p* < 0.001), Supporting H2, with a large standardized effect size. In turn, strategic planning perception significantly predicted coping strategies (*B* = 0.103, *β* = 0.213, SE = 0.030, *t* = 3.482, and *p* < 0.001), Supporting H3, representing a small to moderate standardized effect.

**FIGURE 2:**
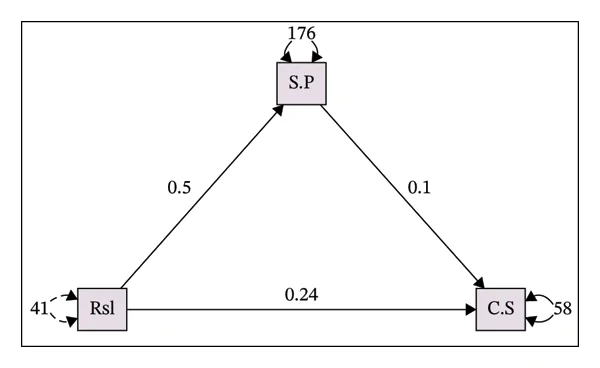
Path analysis of direct and indirect effects of psychological resilience and coping strategies among nursing staff mediated by strategic planning perception. Note: Values represent standardized regression coefficients (*β*); ^∗^ *p* < 0.001; values in parentheses represent *R* ^2^; the indirect effect (*β* = 0.181) represents the effect of resilience on coping transmitted through strategic planning perception.

The indirect effect of psychological resilience on coping strategies through strategic planning perception was statistically significant (*B* = 0.052, *β* = 0.181, SE = 0.018, 95% CI [0.016, 0.088], and *p* < 0.001), confirming the presence of mediation. This indirect effect accounted for approximately 37% of the total effect, indicating partial mediation. The total effect of psychological resilience on coping strategies was also significant (*B* = 0.291, *β* = 0.486, SE = 0.062, *t* = 4.681, *p* < 0.001, and 95% CI [0.169, 0.413]). The confidence intervals for both indirect and total effects were consistent with the corresponding regression coefficients, as the bias‐corrected bootstrap 95% confidence intervals did not include zero, as shown in Table 3 and Figure 2. Overall, the findings support the hypothesized mediation model, confirming H4 that strategic planning perception plays a significant partial mediating role in the relationship between psychological resilience and coping strategies among nursing staff.

**TABLE 3:** Mediating role of strategic planning perception in the relation between psychological resilience and coping strategies among nursing staff (*n* = 379).

	*B*	*β*	CI 95% (LL‐UL)	SE	*t*	*p*	*R* ^2^
Direct effect							
Resilience ⟶ Coping strategies	0.239	0.312	(0.116–0.363)	0.063	3.799	< 0.001^∗∗^	
Resilience ⟶ SPP	0.503	0.521	(0.294–0.711)	0.106	4.729	< 0.001^∗∗^	
SPP ⟶ Coping strategies	0.103	0.213	(0.045–0.161)	0.030	3.482	< 0.001^∗∗^	0.271
Indirect effect							
Resilience ⟶ SPP ⟶ Coping strategies	0.052	0.181	(0.016–0.088)	0.018	3.804	< 0.001^∗∗^	
Total effect							
Resilience ⟶ Coping strategies	0.291	0.486	(0.169–0.413)	0.062	4.681	< 0.001^∗∗^	0.486

*Note: B*: unstandardized regression coefficient; *β*: standardized regression coefficient; *t*: *t*‐test of significance;*p*: probability value; *R* ^2^: coefficient of determination. Model fit parameters: *χ* ^2^ = 32.210, df = 14, and *p* < 0.001; CFI = 0.981; IFI = 0.982; RMSEA = 0.059. Bootstrapped confidence intervals based on 5000 bootstrap samples; ^∗∗^ *p* < 0.001. The indirect effect accounts for approximately 37% of the total effect (0.181/0.486 = 0.372).

Abbreviations: CI, confidence interval; LL, lower limit; SE, standard error; SPP, strategic planning perception; UL, upper limit.

## 4. Discussion

This study examined whether strategic planning perception mediates the relationship between psychological resilience and coping strategies among nursing staff, with the findings confirming the hypothesized mediation model and demonstrating that personal resilience and organizational perceptions jointly shape adaptive stress responses in nurses.

Psychological resilience demonstrated a significant positive association with adaptive coping strategies (*β* = 0.312, *p* < 0.001), Supporting H1 and corroborating established evidence that resilient nurses more frequently employ problem‐focused engagement and emotional regulation when confronting workplace stressors [13, 16]. The effect size indicates that resilience accounts for approximately 10% of the variance in coping behaviors, suggesting meaningful practical significance. This aligns with prior research demonstrating that resilience facilitates cognitive flexibility and positive stress appraisal, enabling constructive engagement with occupational demands [9, 30]. In clinical practice, nurses with higher resilience are better equipped to maintain psychological stability and professional functioning when facing the unpredictable and intense demands of patient care, which is essential for sustaining workforce well‐being and quality of care [1, 11].

Resilience was also strongly associated with strategic planning perception (*β* = 0.521, *p* < 0.001), Supporting H2. This robust relationship suggests that resilient nurses are substantially more likely to interpret organizational initiatives positively, perceiving strategic planning as coherent, relevant, and supportive. This finding is consistent with research showing that resilient individuals demonstrate greater adaptability to organizational change and more optimistic interpretations of workplace structures [50, 51]. In practical terms, resilient nurses may recognize strategic planning as an organizational resource that enhances role clarity, professional direction, and institutional support [23]. The strong association, explaining approximately 27% of variance, suggests that personal resilience significantly shapes how nurses cognitively appraise organizational processes, extending the understanding of resilience beyond personal coping to encompass perceptions of organizational functioning.

Strategic planning perception was significantly associated with coping strategies (*β* = 0.213, *p* < 0.001), Supporting H3. Nurses perceive strategic planning as transparent and participatory report greater adaptive coping, consistent with evidence that organizational clarity reduces uncertainty and enhances psychological safety [25, 28]. However, this effect was notably more modest than the direct resilience‐coping relationship, suggesting that while organizational perceptions are consequential, individual psychological resources exert stronger influence on coping behaviors. This differential effect size has important practical implications: While organizational interventions alone may yield modest improvements in coping, they are most effective when combined with individual‐level resilience‐building efforts. Nevertheless, even modest organizational effects can be practically meaningful at aggregate levels, particularly given the cumulative impact on workforce well‐being across large healthcare organizations [41].

The most integrative finding concerns the mediating role of strategic planning perception, with the significant indirect effect (*β* = 0.181, 95% CI [0.016, 0.088], *p* < 0.001) confirming partial mediation and Supporting H4. This indicates that resilience influences coping not only directly but also through nurses’ cognitive evaluations of strategic planning processes, whereby resilient nurses perceive organizational planning more positively, and this constructive appraisal facilitates adaptive coping. The partial mediation, with the indirect effect accounting for approximately 37% of the total effect, suggests that strategic planning perception is a meaningful but not exclusive pathway. This finding extends prior work by identifying organizational cognition as a specific mechanism linking personal resources to behavioral outcomes. While resilience‐coping relationships are well established [20], the mediating role of strategic planning perception has not been previously examined. The partial mediation also implies that other mechanisms, such as self‐efficacy, emotional intelligence, or social support, contribute to this relationship [9, 22]. The mediation effect highlights that coping is shaped not only by internal resilience but also by how organizational systems are perceived and interpreted, underscoring the importance of integrating personal and structural approaches in workforce resilience interventions.

Our findings align with and extend the broader evidence base on resilience‐enhancing strategies. Limungi et al. [41] synthesized evidence from six studies on resilience interventions among pediatric nurses, identifying mindfulness practices, education workshops, mHealth applications, and Employee Assistance Programs as effective strategies, and critically found that implementation success depends on organizational support, leadership engagement, and integration into workplace culture, factors that correspond directly to our finding that strategic planning perception mediates resilience‐coping relationships. This convergence suggests that individual resilience interventions may achieve greater effectiveness when complemented by transparent organizational communication and participatory planning processes. The implementation challenges identified by Limungi et al. [41], including time constraints, scheduling difficulties, and limited organizational support, further underscore the need for integrated individual–organizational approaches. Our findings provide empirical support for this perspective, demonstrating that organizational perceptions serve as a mechanism through which personal resilience translates into adaptive coping behaviors.

Several alternative explanations merit consideration. The cross‐sectional design precludes causal inference; reverse causality is plausible, whereby adaptive coping may enhance strategic planning perceptions, and reciprocal relationships may also exist, with resilience, strategic planning perception, and coping mutually reinforcing one another over time [28]. Unmeasured confounding variables, such as leadership style, organizational culture, and social support, may influence both strategic planning perception and coping, potentially explaining some of the observed relationships [14, 26]. The modest indirect effect reinforces that strategic planning perception is one of multiple mediating mechanisms, suggesting that comprehensive models incorporating additional organizational and individual factors would provide a more complete understanding of how resilience translates into coping.

This study contributes novel evidence by identifying strategic planning perception as a specific organizational mediator of resilience‐coping relationships. Previous research has focused on general organizational factors such as perceived organizational support or workplace climate [26, 28]. The emphasis on strategic planning, a concrete, modifiable organizational process, provides actionable insights for nursing leadership, as strategic planning can be influenced through communication, participation, and transparency [23, 24]. This distinguishes our findings from studies that identify organizational factors that may be more difficult to change or influence, offering nursing leaders specific, actionable strategies for supporting workforce resilience.

Several limitations should be acknowledged when interpreting these findings. The cross‐sectional design restricts the ability to establish causal relationships among the variables, and temporal precedence cannot be established. Additionally, data were collected from nurses working in a single university hospital in Egypt, which may limit the generalizability of the findings to other healthcare settings, geographic regions, or cultural contexts. The reliance on self‐reported measures may also introduce the potential for response bias, including social desirability bias or common method variance. Furthermore, the modest effect of strategic planning perception on coping suggests that other important factors influencing coping were not captured in our model. Future studies using longitudinal designs, experimental approaches, and multisite samples are recommended to further validate and expand upon these findings, and to establish causal relationships and evaluate the effectiveness of integrated interventions targeting both personal and organizational resources.

## 5. Conclusion

This study identified positive associations between psychological resilience and coping strategies among nursing staff, with strategic planning perception partially mediating this relationship. Nurses with higher resilience tended to report more positive perceptions of organizational strategic planning, and these perceptions were in turn associated with greater use of adaptive coping strategies. These findings indicate that both individual psychological resources and organizational factors are related to coping behaviors in nursing staff. The partial mediation suggests that strategic planning perception represents one pathway through which resilience is associated with coping, though other mechanisms likely contribute. The stronger direct association between resilience and coping, relative to the indirect effect through strategic planning perception, implies that individual psychological resources may have a closer relationship with coping than organizational perceptions.

### 5.1. Implications for Nursing Management

The findings of this study highlight several important implications for nursing management. First, nursing leaders should prioritize the development of nurses’ psychological resilience through targeted interventions such as resilience training programs, mentorship initiatives, and supportive leadership practices. Strengthening resilience can enhance nurses’ ability to adopt adaptive coping strategies when facing the complex demands of clinical practice.

Second, the mediating role of strategic planning perception suggests that nurse managers should actively involve nursing staff in strategic planning processes. Transparent communication about organizational goals, participation in planning activities, and regular feedback mechanisms can improve nurses’ understanding and perception of strategic initiatives. Such engagement may foster a stronger sense of organizational alignment and facilitate more effective coping behaviors. Finally, nursing administrators should integrate organizational planning with workforce well‐being strategies. By ensuring that strategic plans are clearly communicated, participatory, and aligned with frontline clinical realities, healthcare organizations can create supportive work environments that strengthen both individual resilience and collective workforce stability. These managerial approaches may ultimately contribute to improved staff well‐being, retention, and quality of patient care.

## Author Contributions

Wafaa Ahmed Saleh: conceptualization, data curation, investigation, methodology, project administration, writing original draft, and writing–review and editing supervision. Mona Metwally El‐Sayed: conceptualization, data curation, formal analysis, investigation, methodology, project administration, supervision, validation, writing original draft, and writing–review and editing. Eman Mohamed Ahmed Elshazly: conceptualization, data curation, investigation, methodology, writing original draft, and writing–review and editing. Ahmed Abdellah Othman: conceptualization, data curation, investigation, methodology, writing original draft, and writing–review and editing. Aml AbdElaal Mohamed Ali: conceptualization, data curation, investigation, methodology, writing original draft, and writing–review and editing. Boshra Karem Mohamed El‐Sayed: conceptualization, data curation, investigation, methodology, project administration, writing original draft, writing–review and editing, and supervision.

## Funding

The researchers would like to thank the Deanship of Graduate Studies and Scientific Research at Qassim University (https://www.qu.edu.sa) for financial support (QU‐APC‐2026).

## Ethics Statement

All procedures conducted in this study complied with the ethical principles established in the Declaration of Helsinki (DoH, Oct, 2008), ensuring the protection, dignity, and rights of all human participants. The Research Ethics Committee of the Faculty of Nursing at Qena University, Egypt, approved the study. Before commencing data collection, ethical approval was secured from the relevant administrative authorities of the participating healthcare institution to ensure compliance with institutional guidelines and authorize research access. All eligible participants received a clear explanation of the study’s objectives, procedures, potential risks, and anticipated benefits. Written informed consent was obtained, confirming voluntary participation in line with established ethical standards.

Confidentiality and anonymity were rigorously maintained, with no collection or disclosure of personally identifiable information. Participants were informed of their right to refuse or withdraw from the study at any stage without penalty or loss of entitled services. These measures were implemented to safeguard autonomy, beneficence, and nonmaleficence, thereby protecting participants’ rights and well‐being throughout the research process.

## Consent

Please see the Ethics Statement.

## Conflicts of Interest

The authors declare no conflicts of interest.

## Data Availability

The data that support the findings of this study are available from the corresponding author upon reasonable request.
